# Impact of transition from permanent pasture to new swards on the nitrogen use efficiency, nitrogen and carbon budgets of beef and sheep production

**DOI:** 10.1016/j.agee.2019.106572

**Published:** 2019-11-01

**Authors:** A.M. Carswell, K. Gongadze, T.H. Misselbrook, L. Wu

**Affiliations:** Sustainable Agriculture Sciences North Wyke, Rothamsted Research, Okehampton, EX20 2SB, UK

**Keywords:** Balance, Carbon, Grazing, Livestock, Nitrogen use efficiency, Nutrient budgets, Reseed

## Abstract

There is currently much debate around the environmental implications of ruminant farming and a need for robust data on nitrogen (N) and carbon (C) fluxes from beef and sheep grazing systems. Here we use data collected from the North Wyke Farm Platform along with the SPACSYS model to examine the N and C budgets and the N use efficiency (NUE) of grassland swards at different stages of establishment. We assessed the transition from permanent pasture (PP) to a high-sugar grass (HSG), and a mixed sward of HSG with white clover (HSGC), identifying data specifically for the reseed (RS) years and the first year following RS (HSG-T and HSGC-T). Dominant fluxes for the N budget were N offtake as cut herbage and via livestock grazing, chemical-N fertiliser and N leaching at 88–280, 15–177, and 36–92 kg N ha^−1^ a^−1^, respectively. Net primary productivity, soil respiration and C offtake as cut herbage and via livestock grazing at 1.9–15.9, 1.74–12.5, and 0.34–11.7 t C ha^−1^ a^−1^, respectively, were the major C fluxes. No significant differences were found between the productivity of any of the swards apart from in the RS year of establishment. However, NUE of the livestock production system was significantly greater for the HSGC and HSGC-T swards at 32 and 42% compared to all other swards, associated with the low chemical-N fertiliser inputs to these clover-containing swards. Our findings demonstrate opportunities for improving NUE in grazing systems, but also the importance of setting realistic NUE targets for these systems to provide achievable goals for land-managers.

## Highlights

• Grazed beef and sheep production has low efficiencies of nitrogen (N) use.
• Grassland N use efficiency can be enhanced through sward-inclusion of clover.
• Replacing chemical-N fertiliser with biological N fixation did not affect yields.
• During reseeding grasslands become a carbon source.
• After establishment new swards become a carbon sink.

## Graphical abstract

**Figure:**
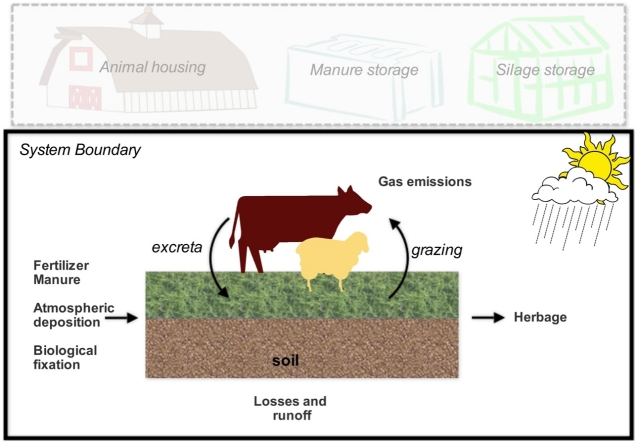

## 1 Introduction

Population growth and changing diets have led to an increased global demand for nitrogen (N) as protein, particularly in animal-based products. Lassaletta et al. (2014) showed that worldwide N consumption grew from 4.1 to 4.6 kg N capita^−1^ year^−1^ between the years 1986 and 2009, with the contribution of animal protein to the human diet increasing from 35 to 39% during the same period. Much of the global growth in protein production has come about through intensifying animal production, which is often dependent upon concentrate feeds sourced beyond the farm-gate. Currently one-third of arable land is used for feed production and based on current consumption patterns this area is expected to increase (Schader et al., 2015). Inclusion of the environmental impacts of N losses in arable systems, in which concentrate feed products are grown, shows large inefficiencies of nutrient use (Bouwman et al., 2013). Consequently, Eisler et al. (2014) suggest that feeding livestock, particularly ruminants, less human edible food is a key step towards achieving sustainable livestock production systems.

Extensive grasslands cover an estimated 26% of the world terrestrial surface and together with permanent pastures amount to 3.5 billion ha globally – more than twice the total area of croplands (FAO, 2019). The grazing of livestock on grasslands not suitable for arable farming can provide a high-quality protein source, from land that cannot otherwise be used directly for human edible food (Schader et al., 2015). Thus, the impact of grasslands with regard to reactive N (Nr) losses to water and air, carbon (C) sequestration and greenhouse gas (GHG) emissions are of global importance as demand for animal-based protein increases (Caro et al., 2014). The soil N and C cycles are tightly coupled and, in the short-term, the soil organic C (SOC) pool can be increased through increased decomposition of plant litter and root material as well as through rhizodeposition (Rees et al., 2005). This can be enhanced through N fertilisation until a new equilibrium is reached (Soussana and Lemaire, 2014). Several studies have shown that managed grasslands can sequester C (Ammann et al., 2007, 2009; Jaksic et al., 2006; Soussana et al., 2004), although the uncertainties around this are high. Additionally, there is a trade-off between the soil C sequestered and the GHG produced during fertiliser production and following fertiliser application (Poulton et al., 2018). Thus, better management of Nr may have a greater impact on C emissions from the agricultural sector than soil C sequestration.

Nutrient budgets are a valuable tool to summarise and understand nutrient cycling in agricultural systems and to assess their environmental impact. In managed grasslands, N and C exports within harvested biomass, as gaseous emissions, via hydrological losses and within the final exported product, can make significant contributions to the system N and C budget. As imbalances are not sustainable in the long term, N and C budgets can be used as indicators and regulatory policy instruments for nutrient management, which can provide insight for methods to reduce losses and increase efficiency. Few studies have attempted to calculate system level N budgets from managed grasslands (Jones et al., 2017; Ma et al., 2012), whereas C budgets have been assessed more often and are available for various ecosystems (Ammann et al., 2007; Felber et al., 2016; Gilmanov et al., 2007; Jones et al., 2017; Kramberger et al., 2015; Siemens and Janssens, 2003; Verlinden et al., 2013). The additional grassland management practice of reseeding has received less attention, especially regarding the impact of reseeding permanent pasture on N and C budgets and system productivity. However, reseeding of grasslands is a common practice. According to a UK-farmer survey (AHDB, 2017) grassland reseeding is used: 1) as a sward-management program; 2) as part of a rotation with arable crops; 3) to counter declining yields; 4) to manage broad-leaf weeds; and 5) to prevent a decline in the prevalence of the sown (and therefore more desirable) grasses.

To provide complete N and C budgets based on monitoring data is generally not feasible because of the difficulty in measuring some of the components. Modelling is an effective way to account for these unmeasured budget components, if model selection is carefully made and as far as possible validated with observed values. Over the last 40 years, crop growth models have been continuously improved to dynamically simulate processes of C, N and water balance with various time-steps to predict crop growth, development and final yield (Basso et al., 2016; Boote et al., 2013; Holzworth et al., 2015; Vereecken et al., 2016). These models integrate multiple processes and consider impacts of environment and management and offer good potential for evaluating farm-scale C and N budgets.

Here, we use the detailed farm-reports for the beef and sheep North Wyke Farm Platform at Rothamsted Research (NWFP; https://nwfp.rothamsted.ac.uk/; see Orr et al., 2016) in conjunction with the SPACSYS model, which simulates simultaneously fluxes exchanged with vegetation and animals, soil and the atmosphere in agricultural systems (Li et al., 2017; Liu et al., 2018; Wu et al., 2007; Wu et al., 2015; Wu et al., 2016). The NWFP farm-reports provided data on livestock movements and weight gain, herbage yield, and field management data including farmyard manure (FYM) and fertiliser inputs and ploughing and reseeding events. Additionally, the farm reports were used to provide input data for SPACSYS, to fully present grassland N and C budgets. With SPACSYS we were able to quantify the relationships among soil nutrients, crop growth and soil water in the soil-plant-atmosphere system. To date, direct measurements of total net primary productivity (NPP) have proved impossible (Ciais et al., 2010), so NPP estimates for grasslands are currently insufficient to produce a pure data-driven estimate. The SPACSYS model is used a useful tool used to determine the net ecosystem CO_2_ exchange (NEE) of intensively managed grasslands by offering an estimation of the C balance, above and below ground C transfer and soil C cycle on a field scale. Thus, we use both monitored and modelled data to examine the impact of the transition from permanent pasture through to the establishment of novel grassland sward-varieties on the system N and C budgets. We apply the N budget data to the 2-dimensional NUE framework presented by the EU Nitrogen Expert Panel (2015) to evaluate the agronomic performance of the transitioning swards relative to permanent pasture. Finally, we explore the environmental impact of grassland reseeding and the establishment of new swards.

## 2 Materials and methods

### 2.1 Site description and experimental design

The NWFP is an experimental platform for beef and sheep production, located in the southwest of England (50°46′N, 3°54′W and 120–180 m a.s.l.). The NWFP has a temperate climate with average annual precipitation of 1008 mm and mean daily minimum and maximum temperatures of 6.9 and 13.8 °C, respectively (data from 2000 to 2016). The site overlays clay shales and the predominant soil type is a Stagni-vertic Cambisol (FAO classification; Harrod and Hogan, 2008), which comprises a slightly stony clay-loam topsoil, overlying a mottled stony clay derived from the carboniferous culm measure. The NWFP soils are described in detail by Harrod and Hogan (2008). The dominant soil series are Halstow and Hallsworth, the Halstow series has moderately firm soil strength, with silt, clay and loam comprising 47, 31 and 12% of the Ap horizon, whereas the Hallsworth series is relatively non-porous, except those pores created by roots, with the Ag horizon comprising 43, 31 and 26% silt, sand and clay. Soil total N and C in the top 10 cm are 4.8 and 46.4 mg kg^−1^ dry weight soil with a C:N of 9.5, soil pH was 5.7 and soil bulk density of 0.8–1.1 g cm^−3^ (https://nwfp.rothamsted.ac.uk/). The site falls within the “Good” grass growth class (AHDB, 2017), making it suitable for dairy, beef and sheep production.

**Fig. 1:**
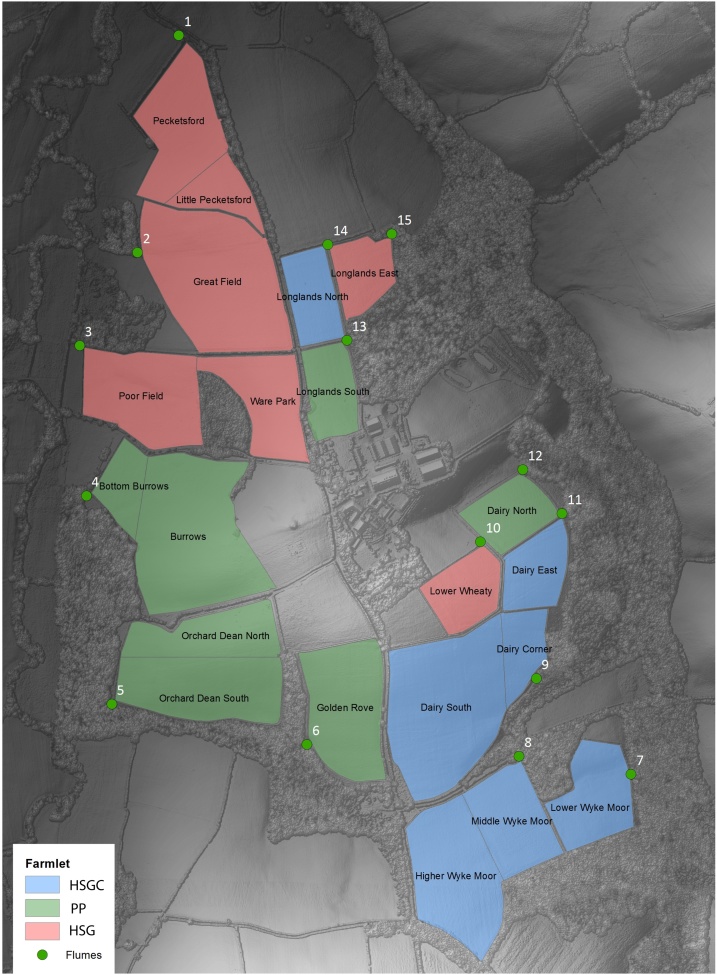
Map of the North Wyke Farm Platform, showing individual fields, subcatchments and flume outlets. Where HSGC = high-sugar grass with clover, PP = permanent pasture, and HSG = high-sugar grass.

We used the NWFP to examine the transition from permanent pasture (PP) to two reseeded swards in comparison with a continuing PP sward, with replication at the subcatchment scale. The different swards are described in full by Orr et al. (2016). In short, the PP sward was maintained as an intensive grassland with regular N fertiliser and FYM amendments. The second sward (HSG) was reseeded with a high-sugar perennial ryegrass (*Lolium perenne* L. Aber^®^Magic), hypothesised to optimise protein utilisation and within-ruminant N partitioning (Miller et al., 2001), and was also managed as an intensive grassland with regular N fertiliser and FYM amendments. The third sward (HSGC) was re-seeded with a mixture of the same high-sugar perennial ryegrass as the HSG treatment together with white clover (*Trifolium repens* L. Aber^®^Herald); consequently, N was provided via biological N fixation (BNF) with additional FYM amendments. Each of the three swards were replicated across 5 hydrologically-isolated subcatchments, equating to a ˜22 ha “farmlet” for each sward (Griffith et al., 2013), and a total of 15 subcatchments (Fig. 1). The transition from the original PP to the new treatments (HSG or HSGC) began in 2013 with the final subcatchments re-seeded in 2015, we present data and simulations from the period 2011 – 2016.

Each farmlet was grazed by 30 beef cattle and 75 ewes with their lambs. Cattle were introduced to the NWFP after weaning, at 6 months of age, and remained on their respective sward treatment until removed for slaughter. Cattle were housed over winter (typically October through to March) and fed grass silage harvested from the individual treatments. Ewes typically grazed longer into the winter season (late November to early January) and were then housed and fed off the NWFP prior to lambing; they were subsequently returned to the NWFP the following Spring (typically March) with their lambs. Average lambing rates were 1.8 for the years 2011 through to 2015, and 1.7 in 2016.

**Fig. 2:**
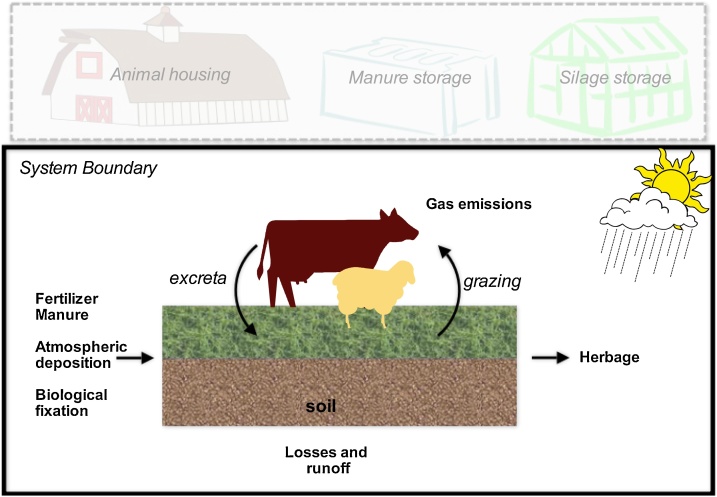
System boundary and fluxes for carbon and nitrogen budgets.

The N and C budgets of the 15 subcatchments were assessed by simulating all relevant input and output fluxes on a calendar year basis (running from 1^st^ January to 31^st^ December). The system boundaries of our approach comprise only the pasture (soil and vegetation) and the animal contribution to the budget by grazing forage and importing excreta during the grazing periods on the investigated subcatchment (Fig. 2). In addition to the N and C budgets animal-N output as a product is also considered for defining livestock NUE.

The data were analysed at the subcatchment scale, whereby subcatchments were categorised as PP for those within the PP farmlet and for all other subcatchments prior to being reseeded (*n* = 60). All subcatchments that underwent ploughing and reseeding with a new sward were categorised as reseed (RS; *n* = 10) for the RS year, as the physical disruption of the reseeding process, the bare ground and sward establishment, was considered the most significant process for those subcatchments within the year. The subcatchments for the year following RS were designated transition (T) years under the new treatments, therefore *n* = 5 for HSG-T (HSG transition) and *n* = 5 for HSGC-T (HSGC transition). After the transition year the new swards were considered established and were designated as HSG and HSGC (*n* = 5 for each). Individual N budgets were derived for each subcatchment (see Table 1. for subcatchment codes) and for each year (from 2011 to 2016). Units for the N budgets were kg N ha^−1^ a^−1^ and for all N terms, the *N balance* was calculated as:

(1) N balance = (N_deposition_ + N_fertiliser_ + N_FYM_ + N_excreta_ + N_fixation_) – (N_intake_ + N_cut_ + N_leaching_ + N_volatilisation_ + N_denitrification_)

Where *N_deposition_* is atmospheric deposition-N (wet and dry combined), *N_fertiliser_* is chemical fertiliser-N, *N_FYM_* is farmyard manure-N, *N_excreta_* is N excreta from grazing livestock including both dung and urine. *N_fixation_* is biological N fixation, *N_leaching_* is hydrological N losses, *N_volatilisation_* is ammonia losses via volatilisation, and *N_denitrification_* is N losses via denitrification.

**Table 1:** Subcatchment codes relating to subcatchment ID and analysis year.

Farmlet	PP 1	PP 2	PP 3	PP 4	PP 5	HSG 1	HSG 2	HSG 3	HSG 4	HSG 5	HSGC 1	HSGC 2	HSGC 3	HSGC 4	HSGC 5
2011	PP	PP	PP	PP	PP	PP	PP	PP	PP	PP	PP	PP	PP	PP	PP
2012	PP	PP	PP	PP	PP	PP	PP	PP	PP	PP	PP	PP	PP	PP	PP
2013	PP	PP	PP	PP	PP	*RS*	PP	PP	PP	*RS*	PP	*RS*	PP	PP	*RS*
2014	PP	PP	PP	PP	PP	HSG-T	*RS*	PP	PP	HSG-T	*RS*	HSGC-T	PP	PP	HSGC-T
2015	PP	PP	PP	PP	PP	HSG	HSG-T	*RS*	*RS*	HSG	HSGC-T	HSGC	*RS*	*RS*	HSGC
2016	PP	PP	PP	PP	PP	HSG	HSG	HSG-T	HSG-T	HSG	HSGC	HSGC	HSGC-T	HSGC-T	HSGC

PP = permanent pasture treatment; *RS* = reseed year; HSG = high-sugar grass treatment, HSGC = High-sugar grass with clover treatment; HSG-T = HSG-transition year; HSGC-T = HSGC-transition year; measurements were made on a yearly-basis running from 1^st^ January to 31^st^ December each year.

Dry matter intake (*DMI*) by grazing animals was estimated by:

(2) DMI = (M_f_ + M_a_ + M_g_)/ME

Where *ME* is the metabolisable energy content of the cut herbage (as MJ kg^−1^ DM); *M_f_*, *M_a_* and *M_g_* are energy requirement for livestock fasting, activity and growth, respectively, and determined by animal average weight (*w;* kg) and liveweight gain (*LWG*, kg d^−1^). A full description of energy requirements can be found in the literature (AFRC, 1993).

Nitrogen intake (*N_intake_*) by animals was divided into two parts: Nitrogen retained by livestock in growth (*N_livestock_*; with growth assumed to be zero for ewes) and N returned to subcatchments via excretion (*N_excreta_*) and estimated by:

(3) N_intake_ = DMI × N_cut%_

Where *N_cut%_* is the measured N content of cut herbage (g N kg^−1^ DM). Nitrogen retained by livestock in growth was calculated by:

(4) N_livestock_ = LWG × A_p_

Where *A_p_* is the protein content of cattle or sheep according to AFRC (1993). All *N_intake_* that was not allocated to growth was defined as *N_excreta_*. N excretion is distributed as dung or urine according to Reed et al. (2015) for cattle (*N_c_dung_* and *N_c_urine_*), and Webb and Misselbrook (2004) for sheep (*N_s_dung_* and *N_s_urine_*):

(5) *N_c_dung_* = [{*Ni_ntake_*×*(*0.345 + 0.317)} / {(*N_intake_*×(14.3 + 0.51))+(*N_intake_* ×(0.345 + 0.317))}] × *N_excreta_*

(6) *N_c_urine_* = [{*Ni_ntake_*×(14.3 + 0.51)} / {(*N_intake_*×(14.3 + 0.51))+(*N_intake_* ×*(*0.345 + 0.317))}] × *N_excreta_*

(7) *N_s_dung_* = *N*_excreta_ × 0.4

(8) *N_s_urine_* = *N_excreta_* × 0.6

Subcatchments were not grazed and the herbage grown was cut and ensiled for feed over winter. This herbage (*N_cut_*) was included in addition to *N_livestock_* as potential livestock output (*N_Plivestock_*) as follows:

(9) N_Plivestock_ = N_livestock_ + {N_cut_ ×(N_livestock_/N_cut_)}

To further assess the agronomic performance of the transitioning swards, the system NUE of both grass production (*NUE_G_*) and livestock production (*NUE_L_*) was derived for each sward treatment at the subcatchment scale as:

(10) NUE_G_ = 100 ×(N_intake_+N_cut_ / N_deposition_+N_fertiliser_+N_FYM_+N_excreta_+N_fixation_)

(11) NUE_L_ = 100 ×(N_Plivestock_ / N_deposition_+N_fertiliser_+N_FYM_+N_fixation_)

### 2.2 Carbon budget

The budget inputs were the net primary productivity (*C_NPP_*), FYM applications and excreta from grazing animals (*C_FYM+excreta_*). Where *C_NPP_* was estimated as:

(12) C_NPP_ = C_GPP_ - C_Ra_

where *C_GPP_* is gross primary production (gross uptake of CO_2_ via photosynthesis) and *C_Ra_* are CO_2_ emissions from autotrophic respiration.

The C outputs were soil respiration (C*_R soil_*), export of organic matter from herbage cuts and animal grazing (*C_intake+cut_*) and leaching of dissolved organic and inorganic C (*C_leaching_*). No data or simulation was available for methane (CH_4_) emissions from enteric fermentation nor animal excretion, therefore CH_4_ emissions from these sources were estimated according to IPCC Tier 2 and Tier 1 methodology respectively (IPCC, 2006; McAuliffe et al., 2018). The sum (balance, ΔSOC) of all C inputs and outputs indicate the storage change in the ecosystem, thus the C budget is calculated as:

(13) ΔSOC / Δt = C_NPP_ + C_FYM+excreta_ – C_Rsoil_ – C_intake+cut_ - C_leaching_

Here we follow the ecological sign convention, in which a negative sign of the overall balance will indicate C loss (C source) and a positive sign will indicate C sequestration (C sink) in the subcatchment soil.

The net ecosystem CO_2_ exchange was defined as:

(14) C_NEE_ = C_Rsoil_ - C_NPP_

Carbon emissions from farm operations has been shown to be less than 1% of system-wide emissions by McAuliffe et al. (2018), therefore this C source was excluded from our C budget.

### 2.3 Data Collation

#### 2.3.1 Empirical data

Livestock were typically weighed every two weeks, with data being used to calculate *w* and *LWG* during each grazing period on a given subcatchment, and an assumption that weight gain was linear between weighing days. Where weight losses were observed, they were adjusted to zero to determine energy requirements.

All subcatchments received N amendments in the form of FYM, and the PP and HSG subcatchments also received chemical N fertiliser. Details of the amount and type of chemical N fertiliser applied were logged in the farm records, as was the amount of FYM applied. Prior to 2014, a standard value of 6 kg N t^−1^ FYM for cattle FYM (Defra, 2009) was used to calculate the N application rate, thereafter FYM samples from each farmlet were analysed for total N (using the Dumas technique) and the measured value was used to calculate the FYM-N application rate.

Fresh herbage yields were determined prior to a silage cut on each subcatchment by cutting five swaths (typically 1.5 x 10 m, using a Haldrup plot harvester) at randomly chosen points within the subcatchment (based on a 25 x 25 m sampling grid). Fresh cut herbage was immediately weighed, and a representative sample taken and dried at 85 °C for a minimum of 24 h, to determine dry matter (DM) content. The DM content of the cut swaths was used to calculate yield on a per hectare basis. Nitrogen content of fresh herbage was determined across the grazing season using fresh snip samples. Snip samples were cut to a residual height of 5 cm following a W-shaped sampling strategy across cattle-grazed subcatchments, and subsequently stored at -18 °C prior to freeze-drying. Dried samples were ground and analysed for total N content using a Carlo Erba NA 2000 linked to a Sercon 20/22 isotope ratio mass spectrometer (Sercon, Crewe, UK; Carlo Erba, CE Instruments, Wigan, UK) and metabolisable energy content following determination of fibres (Givens et al., 2000).

Measurements of ammonia (NH_3_) losses were not included in the NWFP observations, nor in the simulations. Consequently, we applied NH_3_ emission factors to the chemical fertiliser N, FYM and animal urine inputs (*N_c_urine_* and *N_s_urine_*), using UK-specific emission factors as described by Misselbrook et al. (2016), which were 1.8% of chemical N fertiliser, 68.3% of the total ammoniacal N content of FYM (which contributes 10% to total N content of stored FYM; Defra, 2010), and 6% of urine N.

#### 2.3.2 Model simulations

To achieve a better understanding of the N and C cycles in agricultural systems and their storage, we need to be able to accurately measure and model their inputs and losses. This in turn requires a thorough understanding of the biological processes involved and the way in which they are influenced by the physical and chemical environment of the soil. At a field scale, measurements are difficult to obtain, therefore the use of models such as SPACSYS are required to complete, quantitatively, unmeasured elements of N and C budgets. The SPACSYS model (Wu et al., 2007) is a multi-layer, field scale, weather-driven and daily-time-step dynamic simulation model. It includes a plant growth and development component, an N cycling component, a C cycling component, a soil water component for water redistribution through the soil layers, together with a heat transfer component. The main processes concerning plant growth in the model are plant development, assimilation, respiration, and partition of photosynthate and N from uptake, plus N fixation for legume plants, and root growth and development. Nitrogen cycling coupled with C cycling in the SPACSYS model covers the transformation processes for organic matter and inorganic N. The main processes and transformations causing size changes to soluble N pools are mineralization, nitrification, denitrification and plant N uptake. Nitrate is transported through the soil profile and into field drains or deep groundwater with water movement. A biological-based component for the denitrification process to estimate gaseous N emissions was also implemented. The model has been validated with the NWFP data in terms of water fluxes, herbage cuts and soil moisture (Li et al., 2017; Liu et al., 2018; Wu et al., 2016) and with N_2_O emission data collected nearby (Abalos et al., 2016).

The input data required for SPACSYS includes soil properties, weather condition (minimum and maximum temperature, precipitation, sunshine hours), field management (ploughing), plant management (reseeding and cutting dates), fertilizer and FYM application (type, time and amount), and animal management (number of animals and grazing time). All these are freely available on the NWFP data portal (https://nwfp.rothamsted.ac.uk/). Hence, for all 15 fields for the 6-year period, the cut yield biomass was validated with the experimental values. The amount of grazed biomass and excreta was compared and in very good agreement with the approach described in Section 2.2. The simulation outputs were used entirely for the C budget and for the following components of the N budget *N_deposition_*, *N_fixation_*, *N_leaching_*, and *N_denitrification_*.

### 2.4 Statistical analysis

Comparisons were made for the variates *C balance*, C*_NPP_*, *N balance*, *N_cut_+N_intake_, NUE_G_*, and *NUE_L_*, between subcatchments based on the sward present and its stage of establishment as PP, RS, HSG-T, HSGC-T, HSG, and HSGC as shown in Table 1. Subcatchments that were subsequently reseeded were also classified as PP prior to their RS year for the statistical analyses. Unbalanced ANOVA (Genstat v.19, VSN international Ltd) with Fishers unprotected LSD for multiple comparisons was used to examine the variance between sward treatments. Differences were considered significant at the *p* <  0.05 level.

## 3 Results

### 3.1 Nitrogen budget

**Fig. 3:**
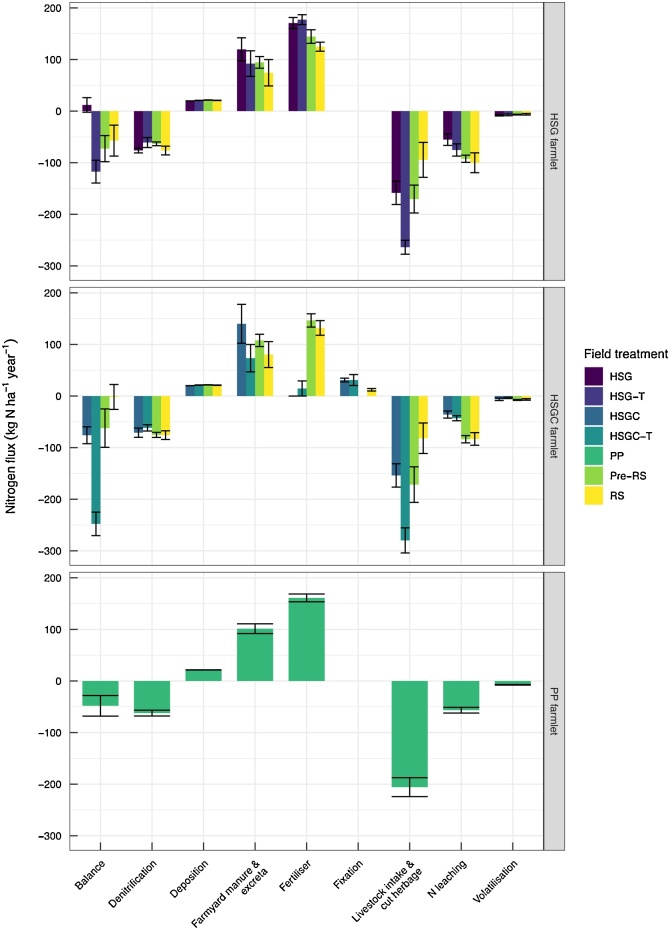
Subcatchment nitrogen budget and balance for each farmlet from permanent pasture to established sward, bars show mean values ± SE. Field treatments are HSG = high-sugar grass; HSG-T = HSG transition year; HSGC = HSG with clover; HSGC-T = HSGC transition year; PP and Pre-RS = permanent pasture (both defined as PP within analysis; and RS = reseed year (RS combined for both the HSG and the HSGC farmlets for the analyses).

Input of total N across all subcatchments and treatments ranged from 140 to 310 kg N ha^−1^ a^−1^ (mean for sward treatments). The major source of N input, excluding HSGC and HSGC-T subcatchments, was chemical-fertiliser N (Fig. 3), which accounted for >50% of total N inputs. Nitrogen from *N_deposition_* accounted for inputs of 21.2 kg N ha^−1^ a^−1^ (± 0.1 SE) across all subcatchments and ranged 19.2–23.5 kg N ha^−1^ a^−1^. Biological N fixation provided another N input in the HSGC and HSGC-T subcatchments where clover was sown, and in five of the ten RS replicates, accounting for 31.0, 31.0 and 12.2 kg N ha^−1^ a^−1^ of N inputs in HSGC, HSGC -T and RS respectively. Together *N_fertiliser_*, *N_FYM_*, *N_deposition_*, and *N_fixation_* supplied an average of 329, 324, 304, 257, 221, and 157 kg N ha^−1^ a^−1^ to HSG, HSG-T, PP, RS, HSGC, and HSGC-T respectively. The other major N input was *N_excreta_* from grazing livestock. Nitrogen excreta was greatest in the HSGC subcatchments at 105 kg N ha^−1^ a^−1^, and accounted for 55% of all N inputs, whereas it was lowest in the RS subcatchments at 38.7 kg N ha^−1^ a^−1^, accounting for just 16% of N inputs.

Total N outputs ranged from 262 to 407 kg N ha^−1^ a^−1^ (mean of sward treatments). The majority of N was removed from the subcatchments as *N_cut_* (Fig. 3) for all treatments, except HSGC, accounting for 61, 58, 56, 46, 43, and 38% of N outputs from PP, RS, HSGC-T, HSG-T, HSG, and HSGC respectively. When *N_intake_* was included as herbage production (*N_cut_* plus *N_intake_*), overall herbage production was greatest in the transitioning swards HSGC-T and HSG-T at 280 and 264 kg N ha^−1^ a-1 and least from the RS sward at 88 kg N ha^−1^ a^−1^, which was statistically similar to HSGC and HSG (*p* <  0.01). After *N_cut_*, *N_intake_* was the second major N output from the subcatchments. However, only a fraction of *N_intake_* was retained as *N_livestock_*, at 9.36, 8.75, 5.33, 4.42, 3.80, and 3.39 kg N ha^−1^ a^−1^ for the HSG, HSGC, HSG-T, PP, HSGC-T, and RS subcatchments respectively. When *N_cut_* was included as additional capacity for sward grazing (as *N_Plivestock_*) the system-livestock production increased to 18.4, 18.0, 14.6, 12.2, 11.6, and 6.3 kg N ha^−1^ a^−1^ for HSG-T, HSGC-T, HSG, HSGC, PP, and RS respectively (*p* =  0.06).

All other N outputs shown (Fig. 3) can be defined as N losses, of which the simulated outputs *N_denitrification_* and *N_leaching_* dominated. Denitrification accounted for 14.9–29.0% of N outputs, and *N_leaching_* losses accounted for 11.1–34.9% of N outputs. Overall, the greatest N outputs were observed from the PP subcatchments, followed by HSG-T, whereas the HSGC subcatchments had the lowest total N outputs (Fig. 3). When N losses were combined with *N_intake_* and *N_cut_* the N outputs generally exceeded N inputs, as shown by the N balance data (Fig. 3). Only the HSG sward had a positive N balance once all inputs and outputs were accounted for at 11.9 kg N ha^−1^ a^−1^, and the HSGC-T had a significantly lower N balance than all other swards at −248 kg N ha^−1^ a^−1^ (*p* =  0.002).

### 3.2 Nitrogen use efficiency

**Fig. 4:**
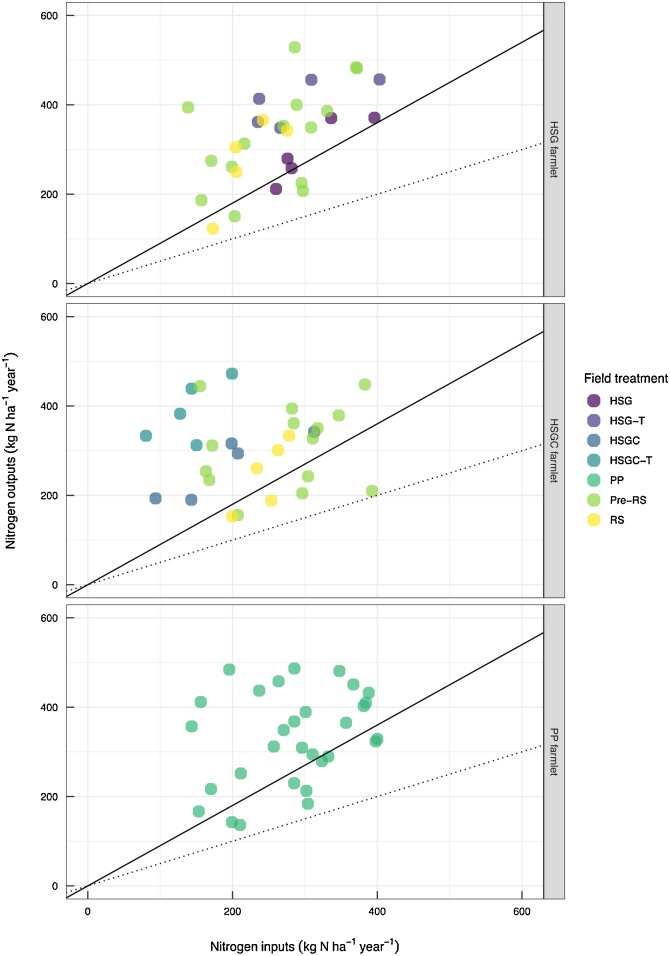
Nitrogen use efficiency (NUE) of grass production system for each farmlet from permanent pasture to established sward, points show individual subcatchment values. Solid line shows NUE of 90% and dotted line shows NUE of 50%. Field treatments are HSG = high−sugar grass; HSG−T = HSG transition year; HSGC = HSG with clover; HSGC−T = HSGC transition year; PP and Pre−RS = permanent pasture (both defined as PP within analysis; and RS = reseed year (RS for both the HSG and HSGC farmlets are combined in the statistical analyses). Differences between NUE of grass production for the field treatments were examined using unbalanced ANOVA, with Fishers LSD for multiple comparisons. NUE superscripts were a, ab, b, b, b, and c for RS, HSG, PP, HSGC, HSG−T, and HSGC−T (*p* = <0.001).

**Fig. 5:**
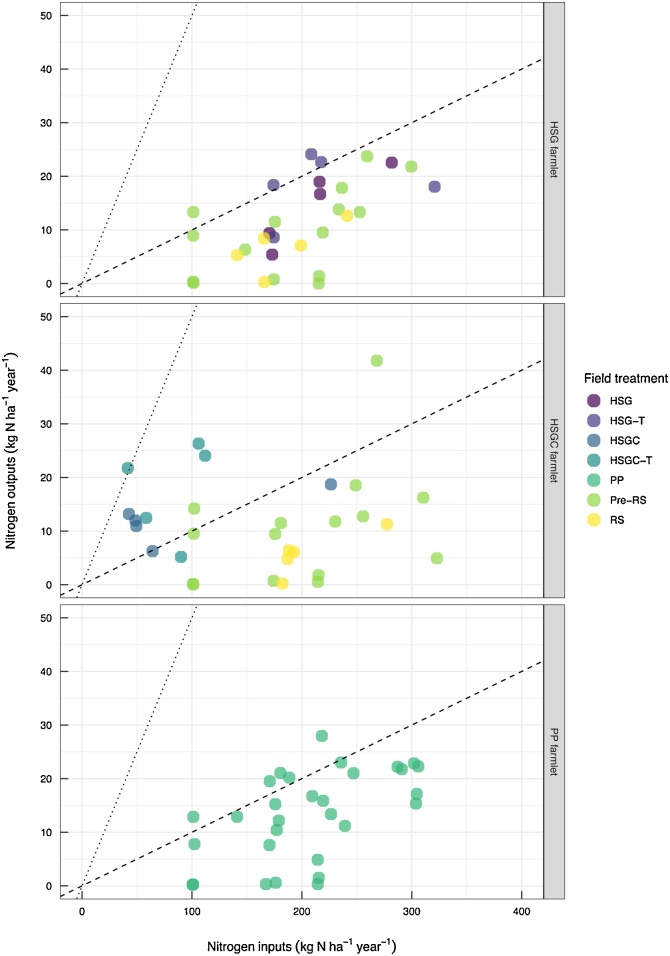
Nitrogen use efficiency (NUE) of the livestock production system for each farmlet from permanent pasture to established sward, points show individual subcatchment values. Dotted line shows NUE of 50% and dashed line shows NUE of 10%. Field treatments are HSG = high−sugar grass; HSG−T = HSG transition year; HSGC = HSG with clover; HSGC−T = HSGC transition year; PP and Pre−RS = permanent pasture (both defined as PP within analysis; and RS = reseed year (RS for both the HSG and HSGC farmlets are combined in the statistical analyses). Differences between NUE of livestock production for the field treatments were examined using unbalanced ANOVA, with Fishers LSD for multiple comparisons. NUE superscripts were a, a, a, a, b, and b for RS, PP, HSG, HSG−T, HSGC and HSGC−T (*p* = <0.001).

*NUE_G_* was extremely variable (Fig. 4), ranging from 40% for the RS treatment to 257% for the HSGC-T treatment. *NUE_G_* was significantly greater (*p* <  0.001) for HSGC-T than for all other treatments, with the RS sward obtaining significantly lower *NUE_G_* values than all other treatments excluding HSG. *NUE_L_* for the different swards through their introduction (RS) and transition to established sward is shown in Fig. 5 on a subcatchment basis. *NUE_L_* was typically very low at <10% for the PP, HSG and HSG-T swards, with an extreme *NUE_L_* of 3.5% observed from the RS subcatchments. In contrast, the HSGC-T and HSGC swards typically obtained *NUE_L_* values > 10%, at 42.2 and 32.4%, significantly greater than for all other swards (*p* < 0.001).

### 3.3 Carbon budget

**Fig. 6:**
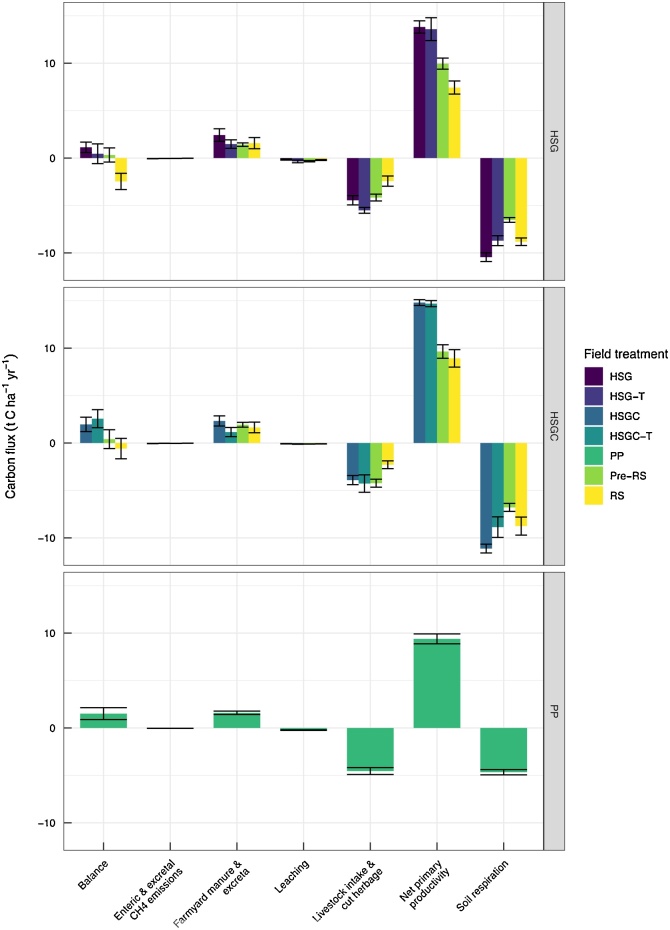
Subcatchment carbon budget and balance for each farmlet from permanent pasture to established sward, bars show mean values ± SE. Field treatments are HSG = high-sugar grass; HSG-T = HSG transition year; HSGC = HSG with clover; HSGC-T = HSGC transition year; PP and Pre-RS = permanent pasture (both defined as PP within analysis; and RS = reseed year (RS combined for both the HSG and the HSGC farmlets for the analyses).

The C inputs considered under NPP ranged from 1.94 to 15.9 t C ha^−1^ a^−1^ for the field treatments, with the maxima observed on HSG and the minima on PP subcatchments (Fig. 6). Average NPP was significantly greater from the HSGC and HSG relative to the PP and RS treatments, at 14.8, 13.8, 9.8 and 8.2 t C ha^−1^ a^−1^ respectively (*p* < 0.001). For most subcatchments, FYM plus excreta contributed 14% toward C inputs, however for the HSG-T and HSGC-T subcatchments FYM plus excreta was 8.5% of C inputs and 16% for the RS years.

Carbon outputs as cut herbage and intake from grazing livestock ranged 2.36–5.51 t C ha^−1^ a^−1^, with RS significantly lower than all other treatments (*p* = 0.007), excluding HSGC which was statistically similar. Leached C and enteric and excretal CH_4_-C losses from all treatments ranged 75 to 353 and 18–60 kg C ha^−1^ a^−1^respectivey. Whereas soil respiration ranged 4.7 - 11.1 t C ha^−1^ a^−1^. For the C balance there were no significant differences between treatments. Generally, only the RS treatment acted as a C source at -1.53 t C ha^−1^ a^−1^, and the remaining treatments were all C sinks.

## 4 Discussion

### 4.1 Grassland N inputs and outputs

Total N inputs varied substantially between treatments, with the greatest mean inputs observed for the HSG treatment at 310 kg N ha^−1^ a^−1^ (*n* = 5), and the least for the HSGC-T treatment at 140 kg N ha^−1^ a^−1^. The range of values reported here are comparable to those reported for dairy systems by Mihailescu et al. (2014) and de Klein et al. (2017) of 118–301 and 229–351 kg N ha^−1^ a^−1^ respectively. Fertiliser N was the greatest N source for the PP, HSG, HSG-T and RS subcatchments at 128–177 kg N ha^−1^ a^−1^, which is greater than the average British values for cattle and sheep at 103 kg N ha-1 (Defra, 2017; on grass < 5 years old). The HSGC and HSGC-T treatments relied on *N_fixation_* and *N_FYM_* as N sources, in addition to the excreta from grazing animals. However, these N inputs combined did not equate to that of N fertiliser on the other treatments, with combined totals of 66 and 46 kg N ha^−1^ a^−1^ for the HSGC and HSGC-T treatments respectively, and *N_fixation_* estimated to provide 31.0 kg N ha^−1^ a^−1^ to these totals. Our simulated *N_fixation_* values are in line with the 40 kg N ha^−1^ reported for an organic dairy farm, which made no chemical fertiliser amendments (Oenema et al., 2012). Excretal returns ranged from 59 to 105 kg N ha^−1^ a^−1^ for the swards examined here, similar to the 31 to 104 kg N ha^−1^ excretal returns of medium-high grazing intensity dairy farms (Oenema et al., 2012), and the mean of 93 kg N ha^−1^ reported by Cherry et al. (2012) across a variety of pasture systems.

Unsurprisingly, the greatest N output from all NWFP treatments was as herbage, either cut (*N_cut_*) or via animal intake (*N_intake_*), with values typically greater than the crop N output reported by Cherry et al. (2012) of 251 kg N ha^−1^ within the same region of England. However, when herbage outputs were converted to animal product (*N_Plivestock_*) the output (meat plus bones) became minor, ranging from 6.3 to 18.4 kg N ha^−1^ a^−1^ across all treatments. Although based on a different method for determining N content of *LWG*, the findings presented here are generally lower than those reported for a PP site in Scotland by Jones et al. (2017) who observed *LWG* N outputs of 13–31 kg N ha^−1^ based on the daily *LWG* of heifers and lambs from 2004-2010. For the NWFP PP treatments, values ranged from 0 to 42 kg N ha^−1^ for individual subcatchments, with the lowest N outputs attributed to fields that were grazed by ewes alone and not primarily used for meat production.

The transition of a grassland sward from PP to a new grass variety or mixed sward and the impact of this on the N balance is highlighted in Fig. 3. Grassland productivity declines substantially during a RS year, which is expected due to a period of bare ground whilst the sward becomes established. However, the trade-off is that the new sward, once established, should become more productive than the sward it replaced. This trade-off seems evident in the subcatchments for the first year following RS, with significantly greater herbage-N production (*N_cut_* plus *N_intake_*) achieved from the HSG-T and HSGC-T subcatchments, which is also reflected in a more negative N balance for the HSG-T and HSGC-T years. However, unlike Hopkins et al. (1990) who observed significantly greater total herbage yields in the first year following reseeding relative to PP, the herbage-N production from HSG-T and HSGC-T were statistically similar to that of the PP subcatchments (Fig. 3), which suggests that the supposed ‘trade-off’ for an unproductive RS year did not occur at the NWFP site. Additionally, once the new swards were considered established, as HSG and HSGC treatments, their overall herbage-N productivity was less than that of the PP, so the productive benefits of reseeding were quickly lost. Our findings also agreed with those of Nevens and Reheul (2003); over thirty-one years they found no difference in average feed energy values between a PP and a three-year ley. However, they suggested that the younger grassland was able to produce similar yields to that of the PP without the need for high levels of N fertiliser (Nevens and Reheul, 2003), as shown here for the HSGC and HSGC-T treatment.

After productive N outputs, the remaining N outputs can be described as N losses, the greatest of these was *N_denitrification_* (N_2_, N_2_O and NO). Studies which include gaseous N losses are scarce, particularly where gas speciation is included. To our knowledge the study by Jones et al. (2017) is the only one that considers the full range of gaseous losses within their N budget, with which we can make comparisons. Their combined N_2_ and N_2_O losses were 14–45 kg N ha^−1^, whereas our denitrification values were greater at 61–76 kg N ha^−1^ a^−1^. However, our NH_3_ losses were lower at 4.1–9.0 kg N ha^−1^ a^−1^ relative to their combined NH_3_ and NO_X_ losses at 36–68 kg N ha^−1^, although this may be due to the inclusion of NO_X_ within the volatilisation estimate of Jones et al. (2017), whereas NO_x_ is included within the *N_denitrification_* output in this study. Leached N losses (*N_leaching_*) were greatest during RS years, highlighting an additional N-loss pathway that should be accounted for when reseeding. Greater N leaching can be attributed to mineralisation of soil organic material following ploughing (Whitmore et al., 1992; Scholefield et al., 1993), and the subsequent mobilisation of N during surface and subsurface flow. Scholefield et al. (1993) reported increased N leaching in the first winter after ploughing and reseeding, however in their study N leaching was reduced in new swards relative to PP by the third year following sward establishment. Our estimated *N_leaching_* losses (which include NH_4_, NO_3_+NO_2_, and dissolved organic N) at 36–92 kg N ha^−1^ a^−1^ were within the range of that reported by Jones et al. (2017) from permanent pasture at 10–149 kg N ha^−1^.

### 4.2 Nitrogen use efficiency

The *NUE_G_* values were extremely variable across management systems, but the mean values of 41–94% for the treatments receiving chemical-N fertiliser compared well to other *NUE_G_* values dependent on chemical-N fertiliser as a major N source, including 55–80% for cut swards (Ball and Ryden, 1984); 56–79% for 15 dairy farms in the Netherlands (Oenema et al., 2012); 58% for a beef and sheep farm in Scotland (Jones et al., 2017); and 66% for 25 pasture-based farms in southwest England (Cherry et al., 2012). The *NUE_G_* for the sward treatments dependent on *N_fixation_* as their N source (HSGC and HSGC-T) was much greater at 87–199%, which at the lower end was comparable to the organic dairy farm included in the study by Oenema et al. (2012) of 91%. However, in accordance with the framework outlined by the EU Nitrogen Expert Panel (2015) NUE values >90% are indicative of soil mining, suggesting that the soil is being mined for N in the HSGC farmlet (Fig. 4) which may be unsustainable in the long-term.

When *NUE_L_* was calculated and N outputs are only those gained within *N_Plivestock_* the *NUE_L_* falls below 50% and is often below 10% (Fig. 5). The HSGC and HSGC-T treatments obtain significantly greater *NUE_L_* than the other treatments at 32 and 42% respectively, and this can be linked to the low N inputs of this management system. However, in terms of productivity the treatments that relied on chemical-N fertiliser did not achieve significantly greater outputs as *N_Plivestock_* than the HSGC and HSGC-T treatments. The NWFP *NUE_L_* values for the treatments receiving chemical-N fertiliser compare well with other values obtained in the literature of 5–17% for beef and sheep (Jones et al., 2017); and 5–20% for meat and milk in grazed systems (Ball and Ryden, 1984). Often when presenting the NUE of ruminant systems, studies present the efficiency of the ruminant for converting feed-N to meat or milk-N, and these NUE values can be greater (15–36%; de Klein et al., 2017) than those presented here, which account for the whole ruminant production system. It should also be noted that we do not take the *NUE_L_* through to full chain NUE which also accounts for N losses at the abattoir, meat processing and food waste stages of food production, as presented by Leach et al. (2012), which would further lower the NUE of livestock production.

The *NUE_L_* values reported here and from elsewhere within the literature demonstrate the need for achievable NUE targets to be set for ruminant systems, so that land-managers can reduce inefficiencies in their systems and have feasible targets to aim for. For this to occur there is a requirement for better understanding of N fluxes at the farm and sub-farm level. This will be dependent on an improved ability to measure N fluxes, particularly herbage and forage growth; offtake and retention by grazing animals, inclusion of the winter housing component of livestock systems, and the recycling of manures. De Klein et al. (2017) suggest that NUE may not be the best metric for determining farm performance, especially where the end-goal is to reduce environmental N losses, instead they suggest that whole-farm N surplus targets would be a better metric for farmers to measure their performance by. The data presented within this study agrees with this recommendation, as NUE can vary significantly depending on N inputs. However, as shown in Fig. 3, substantial N losses to the environment, via leaching and denitrification, can still occur within a low N input system.

### 4.3 Carbon budget

The NWFP subcatchments were a sink for atmospheric CO_2_, according to the SPACSYS simulations, except during the years where ploughing and sowing occurred where the RS subcatchments became a C source (Fig. 6). NPPs of 14.7, 13.7 and 9.8 t C ha^−1^ a^−1^ for HSGC, HSG and PP respectively are within the range reported for pasture system within Europe using PASIM (Ciais et al., 2010). It has been shown that higher net primary production leads to higher C sequestration rates (Ammann et al., 2007; Conant et al., 2001). Our results confirmed this for the HSGC and HSG subcatchments. However, the PP subcatchments may not have reached their potential for C sequestration as even during the new sward-transition years (HSG-T, and HSGC-T) greater values of 13.6 and 14.7 t C ha^−1^ a^−1^ were estimated. This can be linked to the ability of young plants to transfer belowground 50% more of the assimilated C for root construction, maintenance and root respiration (Rees et al., 2005). Hence, the trade-off of C losses during the RS years are quickly balanced within the following year of sward establishment, therefore introduction of innovative high-production swards may enhance C sequestration. For the established HSG and HSGC treatments, annual GPP was estimated at 25.5 and 28.1 t C ha^−1^ a^−1^ and 17.6 and 13.7 t C ha^−1^ a^−1^ for PP and RS, respectively. The GPP values of the HSG and HSGC treatments were close to the upper range of 23 t C ha^−1^ reported for intensively/extensively managed grasslands (Gilmanov et al., 2010; Jones et al., 2017). In a site in Ireland, the opposite trend for GPP was reported, with the permanent pasture producing higher GPP of 29 t C ha^−1^ and 21.4 t C ha^−1^ for a recently established grassland (Byrne et al., 2005). This could be explained by lower tiller density, due to insufficient grass-seeding, and consequently limited grass production (Byrne et al., 2005). The PP value of 17.6 t C ha^−1^ a^−1^ from our study is also slightly lower than values reported for New Zealand dairy pastures of 20.0 t C ha^−1^ (Mudge et al., 2011).

The total respiration, R_total_ was 24, 22.7, 14.4 and 14.35 t C ha^−1^ for HSG, HSGC, PP and RS respectively. The R_total_ was 50% higher for the reseeded treatments, with the highest rate occurring for the HSG treatment where consequently the GPP was also the highest (Byrne et al., 2005). Hence, we can conclude that HSG and HSGC seeding rates were adequate, achieving high tiller density and grass production and maximizing the output potential. Soil respiration is generally greater for grazed pastures than for meadows kept for conserved forage as 20–40% of the ingested C is returned to the soil as dung (Soussana et al., 2004). This extra source of C for soil decomposition, as well as a higher NPP, both contribute to increase soil respiration under grazing compared to cutting. The soil respiration was doubled under the HSG and HSGC treatments too.

The estimated values for NEE were -3.67, -3.37, and - 3.14 t C ha^−1^ a^−1^ for HSG, HSGC and PP respectively all indicating C gain, within the range of 1–6 t C ha^-1^ reported for temperate grasslands (Jones et al., 2017), established permanent pasture (Byrne et al., 2005), extensively managed grasslands around the world (Gilmanov et al., 2010) and intensively grazed temperate pasture in New Zealand (Mudge et al., 2011). The only years when the NEE was positive (0.61 t C ha^−1^) and a net source of CO_2_ was during reseeding. Ploughing and cultivation disturbs soil aggregates, breaks soil structures and increases aeration and rate of decomposition. However, already in the first year of establishment of the new swards, the sink function was restored.

When all components of C input and output were accounted for, average C balance was -1.53, 0.45, 0.94, 1.13, 1.96 and 2.57 t C ha^−1^ for RS, HSG-T, PP, HSG, HSGC and HSGC-T respectively. Interestingly, the C sequestration increased from 0.45 to 1.13 t C ha^-1^ a^-1^ from the HSG-T years to the established HSG years. The opposite trend was observed for the HSGC treatment where C storage decreased from 2.57 to 1.96 t C ha^-1^ a^-1^ from the HSGC-T years to the established HSGC years. These estimated values are within the estimated ranges for European grazed and cut grasslands of 1.04 ± 0.73 t C ha^−1^ (Soussana et al., 2007); a New Zealand dairy farm of 0.59 – 0.90 t C ha^-1^ (Mudge et al., 2011); a Swiss grassland 1.47 ± 1.30 t C ha^-1^ (Ammann et al., 2007); and a Scottish grassland of 1.63 ± 1.40 t C ha^-1^ (Jones et al., 2017).

Due to the C export from cut herbage (subsequently used for winter feed) as well as the stimulation of primary production through grazing, C sequestration tends to be lower in cut compared to grazed systems (Soussana et al., 2004). However, C yielded from herbage-cuts will end up as animal feed; this C will be digested and respired off-site, thus releasing CO_2_ and CH_4_ to the atmosphere as well as being returned to the grassland as manure or slurries.

Our study confirms that C sequestration in grassland has the potential to partly mitigate the GHG balance of ruminant production systems at low grazing intensities (Soussana and Lemaire, 2014). However, grasslands can only act as a C sink until they reach their saturation point. Our findings show that NPP and soil N cycling was increased especially in the legume treatment, leading to greater soil C sequestration. Similarly, the carbon use efficiency (CUE), defined as NPP/GPP, gave the HSGC treatment best value of 0.57 followed by 0.53 (PP) and 0.5 (HSG), all values lying within typical grassland ranges of 0.35 to 0.65 (Amthor and Baldocchi, 2001). Yet, evaluating the NPP use efficiency (as defined by the ratio of exported C to NPP (Vuichard et al., 2007) showed that the PP performed the best with 0.48 followed by 0.32 and 0.26 for HSG and HSGC, respectively. Assuming no overgrazing, the environmental impacts of grassland intensification are therefore controlled by a trade-off between increased coupling of C and N by vegetation and increased decoupling of C and N by animals. Improved grassland management and integration with crop systems may help minimize the harmful environmental effects of C and N decoupling by ruminants, thereby providing environmental, animal welfare, nutritional and food integrity benefits.

## 5 Conclusions

Our findings highlight the inefficiency of N use that can occur within grazed beef and sheep systems receiving chemical-N fertiliser, and the potential for increasing nitrogen use efficiencyin these systems. We were able to demonstrate the highly efficient use of applied N by the grass and grass with clover swards for all transitioning, established and permanent pasture sward treatments, ranging from 54 to 257%. However, this efficiency was markedly reduced when determined for livestock output, ranging from 7 to 42%. Nitrogen use efficiency was much reduced during reseed years, at 3 and 40% for the livestock- and grass-production systems respectively. Although yields increased in the first year following reseeding, we were not able to demonstrate any significant yield improvement for the reseeded treatments relative to the permanent pasture sward. However, grassland reseeding to include legumes within the new sward significantly enhanced nitrogen use efficiency of the livestock production system. Therefore, if the aim of grassland reseeding is to reduce N inputs and dependence on chemical-N fertiliser, then reseeding with legumes could provide a viable grassland management option.

On an annual basis, our simulations suggest that the established swards acted as a sink for atmospheric CO_2_ in most years, with C losses typically occurring during reseed years. Grazing can decrease CO_2_ uptake by plants due to a reduced leaf area index and animal respiration being a source of CO_2_, however the introduction of innovative sward varieties could reverse this effect and deliver higher C sequestration rates. Thus, improved grassland may help minimize the harmful environmental effects of C and N decoupling by ruminants, thereby providing environmental, animal welfare, nutritional and food integrity benefits.
